# Allergen-specific IgE/total IgE ratio for food allergy diagnosis in children

**DOI:** 10.3389/fped.2025.1628506

**Published:** 2025-10-17

**Authors:** Xiao Xu, Ling Liu, Yuedi Zhang, Pengxiang Zhou, Pan Wang, Jiansuo Zhou, Wei Zhou

**Affiliations:** ^1^ Department of Pediatrics, Peking University Third Hospital, Beijing, China; ^2^ Department of Pharmacy, Peking University Third Hospital, Beijing, China; ^3^ Institute for Drug Evaluation, Peking University Health Science Center, Beijing, China; ^4^ Department of Laboratory Medicine, Peking University Third Hospital, Beijing, China; ^5^ Department of Laboratory Medicine, Peking University Third Hospital-Chongli, Zhangjiakou, Hebei, China

**Keywords:** retrospective studies, oral food challenge, immunoglobulin E ratio, food hypersensitivity, child

## Abstract

**Background:**

This study aimed to assess the relationship of allergen-specific IgE (sIgE) levels and the ratio of sIgE to total IgE (sIgE/tIgE) with the results of the oral food challenge (OFC).

**Methods:**

We retrospectively analysed the medical records of children diagnosed with or suspected of having food allergies in the Department of Paediatrics of Peking University Third Hospital between January 2012 and July 2023. Spearman's correlation, receiver operating characteristic (ROC) curves, and logistic regression models were used to compare the sIgE levels, sIgE/tIgE, and OFC results.

**Results:**

Eighty-three children with 209 OFC trials were enrolled in this study; sIgE and tIgE levels were tested in 209 children. Among them, 69 children were tested for egg white allergy, 51 for cow's milk allergy, and 52 for wheat allergy. Using multifactorial logistic analysis, in all the samples, the regression coefficient of sIgE was 0.014 (*p* = 0.1), while that of the sIgE/tIgE was 0.026 (*p* < 0.01; OR = 1.026). In the egg white allergic group, the regression coefficient of sIgE was 0.032 (*p* = 0.26), while that of the sIgE/tIgE was 0.02 (*p* = 0.043; OR = 1.020). No significant differences were observed in the sIgE level or sIgE/tIgE between the cow's milk and wheat allergic groups.

**Conclusions:**

The diagnostic value for food allergy sIgE/tIgE ratios, in the total sample and egg white group was better than that of sIgE alone; however, no significant differences were observed in the cow's milk and wheat allergic groups. Further studies with larger sample size or controlled studies are needed to validate these results.

## 1 Introduction

The prevalence of food allergies is increasing and has become a global public health concern (1). Immunoglobulin E (IgE)-mediated food allergies have received widespread attention because of their rapid progression and the risk of severe allergic reactions, which may be life-threatening. Therefore, diagnosing food allergies correctly is important. The oral food challenge (OFC) is the most reliable diagnostic method for food allergies and is recommended by many national guidelines (2), However, OFCs are difficult to promote in routine clinical practice because of its cumbersome process and needs to be performed by allergists. Recently, researchers have been looking at ratio analysis to find a reliable, *in vitro* diagnostic index, to replace the OFC or predict the risk of severe allergic reactions during the OFC to improve its safety.

IgE, a classical marker of type-2 inflammation, plays an important role in the pathogenesis of pediatric allergic disease. Allergen-specific IgE (sIgE) has a high sensitivity for predicting food allergies but lacks satisfactory specificity (3). Positive sIgE results represent sensitisation and should be combined with a history of food allergy to diagnose food allergy. In respiratory diseases associated with pollen allergy, the ratio of sIgE to total IgE (sIgE/tIgE) improves diagnostic sensitivity and specificity (4, 5). However, few studies on the use of sIgE/tIgE ratio for food allergy diagnosis exist. To the best of our knowledge, this is the first study in China that aimed to analyse the role of sIgE/tIgE ratio in food allergy diagnosis in children and compare the results with those of the OFC, considered the gold standard for diagnosis of food allergy, to explore the value of its clinical application.

## 2 Methods

### 2.1 Study population

This was a retrospective, non-interventional study. We reviewed the medical records of children who visited the Department of Paediatrics of Peking University Third Hospital between January 2012 and July 2023 with or suspected of having a food allergy. Data on sex, age, OFC results, food type, sIgE/tIgE results were collected. The results for sIgE and tIgE levels were measured within six months (180 days) before the OFC. Data were collected from 166 children who underwent 365 OFC's. Patients lacking data on sIgE level, tIgE level, or OFC results were excluded. Finally, 83 children with 209 OFC results were enrolled in this study. The study protocol was approved by the Ethics Committee of the Peking University Third Hospital (approval number: 2022 No. 506-01), which waved the requirement for informed consent.

### 2.2 Ige testing

sIgE and tIgE were detected using the Phadia 250 analyser (Thermo Fisher Scientific Co., China) utilising ImmunoCAP® technology. For sIgE, the low and high cut-off values were 0.1 kU/L and 100 kU/L, respectively. Any result >100 kU/L was recorded as 100 kU/L. For tIgE, the low and high cut-off values were 0 and 5,000 kU/L, respectively. Any result >5,000 kU/L was recorded as 5,000 kU/L. Some children underwent repeated sIgE tests; however, the interval between two consecutive tests was >6 months.

### 2.3 OFC

An open OFC was used in this study. The OFC was performed following the procedures in the “Expert Consensus on Standardised Procedures for Oral Food Challenge”, which was based on national and international literature. The OFC was performed by qualified workers from the author's department with experience in conducting OFC tests. Because of individual differences in the duration of cow's milk, egg white, and wheat allergies, some children in this study underwent repeated OFC's to determine food tolerance. Therefore, the number of OFC's performed was higher than the number of children.

### 2.4 Calculation of sIgE/tIgE ratio


Ratio=sIgE×1000/tIgE

### 2.5 Statistical analysis

Categorical variables are expressed as numbers (percentages), and continuous variables are expressed as medians [ranges]. Analyses were performed using the SPSS statistical package (version 22.0; IBM Corporation, Armonk, NY).

Nonparametric tests were applied to compare the differences in sIgE, tIgE, and ratios among different subgroups of OFC results; Spearman correlation analysis and ROC curves were applied to verify the consistency and diagnostic value of sIgE, sIgE/tIgE, and OFC results. Logistic regression modeling was used to assess the relationship between sIgE levels and sIgE/tIgE on OFC outcomes. Statistical significance was defined as *p* < 0.05.

## 3 Results

### 3.1 Patient characteristics

In this study, 83 children were enrolled. Among them, 209 OFC results and 209 serum sIgE and tIgE measurements were obtained. Results about OFC were obtained for 69 tests for egg white, 51 for milk, 52 for wheat, 17 for peanuts, 4 for shrimp, and 1 for crab. The patient characteristics are described in Table 1.

**Table 1:** Summary of patient characteristics, IgE levels and sIgE/tIgE.

Type of food	All food	Egg	Milk	Wheat
Challenges, *n* (%)	209	69 (33)	51 (24.4)	52 (24.9)
Median age at OFC, months	43.5 [3,228]	36 [4,156]	36 [3,132]	45 [5,168]
Sex, *n* (%)				
Male	147 (70.3)	43 (62.3)	33 (64.7)	47 (90.4)
Female	62 (29.7)	26 (37.7)	18 (35.3)	5 (9.6)
sIgE, KU/L				
All sample	3.27 [0.100]	2.59 [0,94.5]	3.14 [0.100]	8.45 [0.02,100]
OFC-positive	9.45 [0,100]	3.15 [0,94.5]	15.1 [0.1,100]	48.5 [0.4,100]
OFC-negative	1.81 [0,100]	2.07 [0.01,43.1]	0.57 [0,11.9]	2.15 [0.02,78.7]
*p* value	<0.01	0.02	<0.01	<0.01
tIgE, KU/L				
All sample	361 [5.45, 5,000]	238 [5.45, 5,000]	159 [11.9, 5,000]	432.5[11.9, 5,000]
OFC-positive	386.8 [5.45, 3,826]	310 [5.45, 1,988]	361 [11.9, 3,826]	465 [11.9, 1,988]
OFC-negative	330 [17.1, 5,000]	238 [18.3, 5,000]	155 [17.1, 5,000]	333 [18.3, 5,000]
*p* value	0.26	0.57	0.88	0.41
sIgE/tIgE ratio				
All sample	12.8 [0, 395.8]	13.5 [0, 94.5]	17.0 [0, 382.2]	44.4 [0, 382.2]
OFC-positive	44.2 [0, 395.8]	31.6 [0, 352.4]	36.3 [2.52, 382.2]	94.8 [1, 325.9]
OFC-negative	4.2 [0, 104.8]	5.1 [0.3, 86.6]	1.4 [0, 72.1]	4.78 [1.09, 4.78]
*p* value	<0.01	<0.01	<0.01	<0.01

Among the 209 cases of obtained results, the median age was 43.5 months (ranging from, 3–228 months). Among the 69 cases of egg protein results, the median age was 36 months (ranging from 4 to 156 months). Among the 51 cases of milk protein results, the median age of 36 months (ranging from 3 to 132 months), while the median age in the 51 cases of obtained test results for wheat allergy was 45 months (ranging from 5 to 168 months).

Among the 209 cases, the OFC results were positive in 111 cases (53.1%) and negative in 98 cases (46.9%). The results were positive in 44 cases (63.8%) and negative in 25 (36.2%) in the egg white group; positive in 29 cases (56.9%) and negative in 22(43.1%) in the milk group; and positive in 29 cases (55.8%) and negative in 23(44.2%) in the wheat group.

sIgE, tIgE, and sIgE/tIgE ratios results also were described in Table 1.

The median sIgE level for all samples was 3.27 kU/L (0–100 kU/L), tIgE of 361 kU/L (5.45–5,000 kU/L), sIgE/tIgE of 12.8 (0–395.8 kU/L); the median sIgE level for egg white group was 2.59 kU/L (0–94.5 kU/L), tIgE was 283 kU/L (5.45–5,000 kU/L), and sIgE/tIgE was 13.5 (0–352.4); the median sIgE level for milk group was 3.14 kU/L (0–100 kU/L), tIgE was 159 kU/L (11.9–5,000 kU/L), and sIgE/tIgE was 17.0 (0–382.2).; the median sIgE in the wheat group was 8.45 kU/L (0.02–100 kU/L), tIgE was 432.5 kU/L (11.9–5,000 kU/L), and sIgE/tIgE was 44.4 (0–382.2).

The tIgE levels were not statistically different in the negative and positive OFC result groups, but both sIgE and sIgE/tIgE ratio suggested a statistically significant difference by nonparametric tests. However, this method was not able to compare the differences between sIgE and ratios.

### 3.2 Comparison of correlation test and diagnostic value of sIgE, sIgE/tIgE, and OFC results

#### 3.2.1 All samples

**Figure 1:**
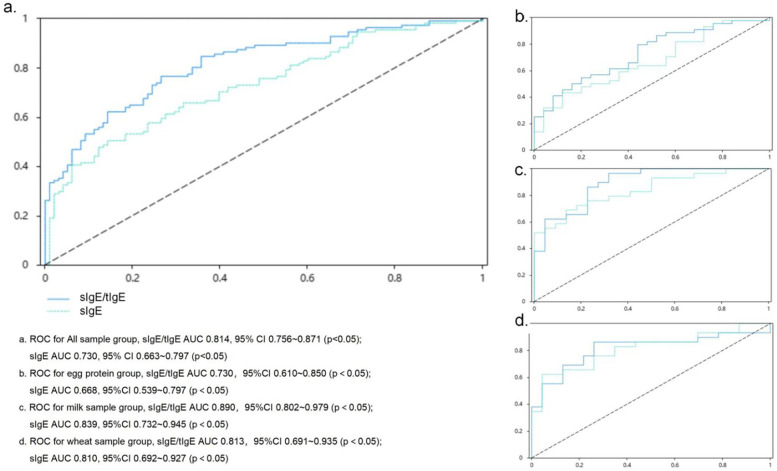
ROC curve assessing the relationship of the sIgE level and sIgE/tIgE ratio with oral food challenge results sIgE, allergen-specific IgE. **(a)** ROC for All sample group, sIgE/tIgE AUC 0.814, 95% CI: 0.756–0.871 (*p* < 0.05); sIgE AUC 0.730, 95% CI: 0.663–0.797 (*p* < 0.05). **(b)** ROC for egg protein group, slgE/tlgE AUC 0.730, 95% CI: 0.610–0.850 (*p* < 0.05); sIgE AUC 0.668, 95% CI: 0.539–0.797 (*p* < 0.05). **(c)** ROC for milk sample group, sIgE/tIgE AUC 0.890, 95% CI: 0.802–0.979 (*p* < 0.05); sIgE AUC 0.839, 95% CI: 0.732–0.945 (*p* < 0.05). **(d)** ROC for wheat sample group, sIgE/tIgE AUC 0.813, 95% CI: 0.691–0.935 (*p* < 0.05); sIgE AUC 0.810, 95% CI: 0.692–0.927 (*p* < 0.05).

Correlation analyses were performed for sIgE, sIgE/tIgE ratio, and the OFC results. The correlation coefficient between sIgE and the OFC results was 0.398 (*p* < 0.05), and that between the sIgE/tIgE ratio and the OFC results was 0.542 (*p* < 0.05). ROC curve analyses showed that the sIgE level corresponded to an area under the ROC curve (AUC) of 0.730 (*p* < 0.05) and an optimal cut-off value of 0.362 (sensitivity, 0.505; specificity, 0.857), while the ratio value corresponding to an AUC of 0.814 (*p* < 0.05) and an optimal cut-off value of 0.500 (sensitivity, 0.766; specificity, 0.735). The sIgE level and sIgE/tIgE ratio had high diagnostic values for the OFC results. A comparison of the AUC between the two groups showed no significant difference between the two methods (Figure 1a).

#### 3.2.2 Egg white group

The correlation coefficient between sIgE level and the OFC results was 0.279 (*p* < 0.05), and that between the sIgE/tIgE ratio and OFC results was 0.383 (*p* < 0.05). ROC curve analysis showed that the sIgE value corresponded to an AUC value of 0.668 (*p* < 0.05), which was of low diagnostic value for OFC results. The ratio value corresponded to an AUC value of 0.730 (*p* < 0.05), which was of limited diagnostic value for OFC results, and the corresponding optimal cut-off value was 0.355 (sensitivity, 0.795; specificity, 0.560). A comparison of the differences in the AUC between the two groups showed no significant differences (Figure 1b).

#### 3.2.3 Cow's milk group

The correlation coefficient between sIgE level and the OFC results was 0.581 (*p* < 0.05), and that between the sIgE/tIgE ratio and the OFC result was 0.670 (*p* < 0.05). ROC curve analyses were performed, and the AUC value corresponding to the sIgE level was 0.839 (*p* < 0.05), which corresponded to an optimal cut-off value of 0.553 (sensitivity, 0.690; specificity, 0.864). The AUC value corresponding to the sIgE/tIgE was 0.890 (*p* < 0.05), which corresponded to an optimal cut-off value of 0.647 (sensitivity, 0.966; specificity, 0.682). The sIgE level and sIgE/tIgE ratio had high diagnostic values for the OFC results. A comparison of the AUC between the two groups showed no significant differences (Figure 1c).

#### 3.2.4 Wheat group

The correlation coefficient between sIgE level and the OFC results was 0.533 (*p* < 0.05), and that between the sIgE/tIgE and the OFC results was 0.538 (*p* < 0.05). ROC curve analyses showed that the AUC value corresponding to the sIgE level was 0.810 (*p* < 0.05), which corresponded to an optimal cut-off value of 0.577 (sensitivity, 0.621; specificity, 0.957). The AUC value corresponding to the sIgE/tIgE was 0.813 (*p* < 0.05), which corresponded to an optimal cut-off value of 0.601 (sensitivity, 0.862; specificity, 0.739). The AUC value corresponding to the sIgE/tIgE was 0.538 (*p* < 0.05) (sensitivity, 0.862; specificity, 0.739). The sIgE level and sIgE/tIgE had high diagnostic values for the OFC results. A comparison of the AUC between the two groups showed no significant differences (Figure 1d).

### 3.3 Predictive value of sIgE and sIgE/tIgE for OFC results

#### 3.3.1 All samples

The sIgE level and sIgE/tIgE were used as independent variables, and the OFC results were substituted into a single factor logistic regression analysis as the dependent variable to explore the predictive value of the two examination methods on the OFC results. The regression coefficient of the sIgE level was 0.038 (*p* < 0.05), and the OR was 1.039 (95% CI: 1.022–1.056) and the regression coefficient of sIgE/tIgE was 0.032 (*p* < 0.05), and the OR was 1.032 (95% CI: 1.020–1.045), suggesting that the sIgE level and sIgE/tIgE among all the samples had a significant positive influence on the OFC results and could predict the OFC results to an extent (Table 2).

**Table 2:** Results of single-factor logistic analysis.

Type of food	sIgE and ratio	Regression coefficient	Standard error	z-value	Wald’s *χ*^2^	*p*-value	OR	95% CI
All samples	sIgE	0.038	0.009	4.483	20.097	0.000	1.039	1.022–1.056
	sIgE/tIgE	0.032	0.006	5.286	27.938	0.000	1.032	1.020–1.045
Egg	sIgE	0.050	0.030	1.662	2.763	0.096	1.052	0.991–1.116
	sIgE/tIgE	0.024	0.010	2.359	5.566	0.018	1.024	1.004–1.045
Milk	sIgE	0.170	0.071	2.396	5.743	0.017	1.185	1.031–1.361
	sIgE/tIgE	0.065	0.025	2.612	6.825	0.009	1.067	1.016–1.120
Wheat	sIgE	0.042	0.013	3.154	9.945	0.002	1.043	1.016–1.070
	sIgE/tIgE	0.026	0.008	3.175	10.078	0.002	1.026	1.010–1.043

tIgE, Total IgE; sIgE, Serum-specific IgE

Furthermore, when the sIgE level and sIgE/tIgE were used as independent variables and the OFC results were substituted into a multifactorial logistic regression analysis as the dependent variable, the regression coefficient of the sIgE level was 0.014; however, no significant difference was observed, suggesting that the sIgE value cannot predict the OFC results very well. Meanwhile, the regression coefficient of the sIgE/tIgE was 0.026 (*p* < 0.05), and the OR was 1.026, implying that the sIgE/tIgE has a significant positive influence on the OFC results, and when the sIgE/tIgE was increased by one unit, the odds of obtaining a positive OFC result were 1.026 times higher (Table 3).

**Table 3:** Results of multifactor logistic analysis.

Type of food	sIgE and ratio	Regression coefficient	Standard error	z-value	Wald’s *χ*^2^	*p*-value	OR	95% CI
All samples	sIgE	0.014	0.009	1.593	2.538	0.111	1.015	0.997–1.033
	sIgE/tIgE	0.026	0.007	3.934	15.478	0.000	1.026	1.013–1.040
Egg	sIgE	0.032	0.029	1.126	1.267	0.260	1.033	0.976–1.093
	sIgE/tIgE	0.020	0.010	2.025	4.102	0.043	1.020	1.001–1.040
Milk	sIgE	0.097	0.066	1.455	2.118	0.146	1.102	0.967–1.255
	sIgE/tIgE	0.038	0.027	1.440	2.072	0.150	1.039	0.986–1.095
Wheat	sIgE	0.023	0.014	1.604	2.571	0.109	1.023	0.995–1.052
	sIgE/tIgE	0.016	0.009	1.815	3.296	0.069	1.016	0.999–1.033

tIgE, Total Ig; sIgE, Serum-specific IgE

#### 3.3.2 Egg white group

The regression coefficient of the sIgE level in the one-way logistic regression analysis of egg protein was 0.050 (*p* > 0.05). The regression coefficient of the sIgE/tIgE was 0.024 (*p* < 0.05), and the OR was 1.024, indicating a significant positive relationship with the OFC results; when the sIgE/tIgE was increased by one unit, the odds of obtaining a positive OFC result were 1.024 times higher (Table 2).

When the sIgE level and sIgE/tIgE were used as independent variables, and the OFC results were substituted into the multifactorial logistic regression analysis as the dependent variable, the sIgE level did not predict the OFC results meaningfully. The regression coefficient of the sIgE/tIgE was 0.020 (*p* < 0.05), the OR was 1.020, and the sIgE/tIgE had a positive influence on the OFC results. When the sIgE/tIgE value was increased by one unit, the odds of obtaining a positive OFC result were 1.020 times higher (Table 3).

#### 3.3.3 Cow's milk group

In the one-way logistic regression analysis in the milk group, the regression coefficient of the sIgE level was 0.170 (*p* < 0.05), and the OR was 1.185 (95% CI:1.031–1.361). The regression coefficient of the sIgE/tIgE was 0.065 (*p* < 0.05), and the OR was 1.067 (95% CI: 1.016–1.120), suggesting that the sIgE level and sIgE/tIgE had a significant positive effect on the OFC results and could predict the OFC results to an extent (Table 2). In the multifactorial logistic regression analysis, neither the sIgE level nor the sIgE/tIgE was significant (*p* > 0.05) (Table 3).

#### 3.3.4 Wheat group

In the one-way logistic regression analysis in the wheat group, the regression coefficient of the sIgE level was 0.042 (*p* < 0.01), and the OR was 1.043 (95% CI: 1.016–1.070). The regression coefficient of the sIgE/tIgE was 0.026 (*p* < 0.01), and the OR was 1.026 (95% CI: 1.010–1.043), suggesting that the sIgE level and sIgE/tIgE had a significant positive influence on the OFC results and could predict the OFC results to an extent (Table 2). When analysed using multifactorial logistic regression, the results of both the sIgE levels and sIgE/tIgE were not significant (*p* > 0.05) (Table 3).

## 4 Discussion

The OFC is a reliable means of diagnosing food allergies; however, the process is relatively cumbersome and needs to be performed by allergists. Children with positive OFC results are at a high risk of rapid or severe allergic reactions. Although fatal incidents caused by OFCs have rarely been reported, a case of death in a patient who had undergone an OFC was reported in 2017 (6), serving as a wake-up call for allergists to emphasise the safety of the OFC. Recently, *in vitro* diagnostic methods have become a popular research topic worldwide (2). Researchers are trying to find more suitable risk assessment indicators before OFC to avoid serious allergic reactions during the OFC process and reduce the difficulty of diagnosing food allergies. Or search for more suitable indicators to evaluate whether children diagnosed with IgE mediated food allergies can undergo OFC, assess whether such foods can be reintroduced, and optimize the management process of food allergies.

The sIgE level has high sensitivity but lacks satisfactory specificity in predicting food allergies, and a comprehensive study with a clear history of food allergies is necessary for its clinical application. We propose sIgE/tIgE because when the specificity ratio of a particular IgE antibody is higher, the surface density of IgE antibody molecules on mast cells and basophils with the same allergen specificity is higher; therefore, the likelihood of inducing mediator release upon encountering the allergen is higher. This ratio more accurately reflects the specific binding capacity on the surface of mast cells and basophils and the likelihood of allergen cross-linking and subsequent activation (7). This reduces the rate of false-positive detections that may be associated with non-IgE and confounding immunolabelling, which is not present in the assay.

Analysis of the sIgE/tIgE can determine food allergenicity, and this ratio is more valuable in patients with very low (<20 kU/L) or high tIgE values (8). In this study, we analysed the sIgE/tIgE with OFC results and verified that this ratio may have a better predictive value for OFC positivity than sIgE alone does.

Herein, we found that sIgE levels and the sIgE/tIgE were significantly positively correlated with the OFC results, suggesting that sIgE levels and the sIgE/tIgE were predictive of the OFC test results. However, via ROC analysis, the AUC corresponding to the sIgE/tIgE was not significantly different from that corresponding to the sIgE level when all foods were analysed together or when a single food group, such as egg white, milk, or wheat, was analysed. The OFC should still be the gold standard for diagnosing food allergies.

Whether it is possible to predict OFC outcomes using *in vitro* tests, determining OFC safety, and choose the appropriate timing for an OFC was also assessed in this study. Gupta et al. (9). found that sIgE/tIgE could predict OFC outcomes. Multifactorial regression analysis in the present study suggested that the predictive value of sIgE/tIgE for OFC outcomes was better than that of sIgE alone in the total sample and egg allergic groups; however, no significant results were obtained for single samples, such as milk and wheat. This is inconsistent with the findings of a study involving 501 children with 992 cases of OFC results (10), which revealed a large discrepancy between the median age of the present study's participants and those reported in the literature. Further refinement of the age subgroups may be needed to validate our results in subsequent studies.

We explored the optimal cut-off value between the sIgE/tIgE and the predicted outcome of the OFC and found that the positivity rate of OFCs was higher when the sIgE/tIgE was >10.42. This suggests that physicians should be cautious in scheduling OFCs when the sIgE/tIgE exceeds 10.42, or they should be more prepared to provide adequate treatment in the event of an OFC to improve safety. For IgE-mediated food allergies, such as those to milk and eggs, a degree of tolerance occurs with age (11, 12), and a decrease in sIgE level may indicate the onset of food tolerance (13). The sIgE/tIgE may be of value in assessing food tolerance and should be investigated in subsequent studies.

Current research suggests that there is no single biomarker that can accurately predict clinical issues such as whether there is an allergy, multiple allergic reactions, or natural remission. However, combining several potential biomarkers can significantly improve the accuracy of allergy disease diagnosis and risk stratification. The classic process of diagnosing food allergies includes a positive medical history, skin prick test, positive results of food specific IgE, and if necessary, assessing the need for OFC based on risk and diagnostic value, and ultimately determining the diagnosis based on the OFC results. The diagnostic value of sIgE/tIgE is discussed in this article, and the results suggest that to some extent, the ratio can predict positive results of OFC or indicate the risk of OFC.

The currently widely recognized potential allergy markers and their detection value include: the value of changes in trypsin in diagnosing severe allergic reactions, the diagnostic value of basophil activation test(BAT) in food allergies, and the diagnostic value of allergen component detection in food allergies or cross allergic reactions (14). But the above tests cannot be conducted in every diagnostic institution. It seems that the clinical diagnostic value of the ratio is not superior to the potential biomarkers mentioned above, but the ratio still has the advantages of being easy to obtain and having certain significance for predicting OFC positive results and risk assessment. But clear results may still require further expansion of the sample size for research.

This study had certain limitations. It was a single-centre retrospective study with a small sample size, lack of detailed differentiation of food characteristics, and lack of grouping according to age. A larger sample size and controlled studies are necessary to validate our results.

In conclusion, the sIgE/tIgE show some promise as a diagnostic and predictive tool for food allergies, particularly in assessing OFC outcomes. Despite the need for further validation with larger cohorts, these findings suggest the potential of the sIgE/tIgE to enhance food allergy diagnosis and management. Future studies should validate the diagnostic and predictive capabilities of the sIgE/tIgE in larger, diverse cohorts.

## Data Availability

The raw data supporting the conclusions of this article will be made available by the authors, without undue reservation.
